# Sporadic isolated congenital asplenia with fulminant pneumococcal meningitis: a case report and updated literature review

**DOI:** 10.1186/s12879-017-2896-5

**Published:** 2017-12-18

**Authors:** Shigeo Iijima

**Affiliations:** Department of Pediatrics, Hamamatsu University School of Medicine, Hamamatsu, Japan

**Keywords:** Sporadic asplenia, Isolated congenital asplenia, Pneumococcal meningitis, Howell-jolly body

## Abstract

**Background:**

Isolated congenital asplenia (ICA) is a rare and life-threatening condition that predisposes patients to severe bacterial infections. Most of the reported cases are familial and the mode of inheritance is usually autosomal dominant. Here, we report a case of sporadic isolated asplenia and review the literature while focusing on sporadic cases.

**Case presentation:**

We report the case of an 11-month-old female infant who developed fulminant pneumococcal meningitis. The pneumococcal vaccine-unimmunized patient was hospitalized with fever, irritability, and purpura, and was diagnosed as having meningitis, septic shock, and disseminated intravascular coagulation. *Streptococcus pneumoniae* was isolated from both cerebrospinal fluid and blood. She was successfully treated with prompt antibiotic therapy. During hospitalization, abdominal ultrasonography and computed tomography findings, scintigraphy results, and Howell-Jolly body-containing red blood cells indicated the presence of asplenia without any visceroarterial anomalies. Moreover, the findings of peripheral blood smears and spleen ultrasonographic examinations of her parents were normal.

**Conclusions:**

Majority of sporadic ICA cases were detected only after the onset of overwhelming infection and had a high mortality. In cases of severe invasive pneumococcal disease, a systematic search for Howell-Jolly bodies on blood smears and the presence of asplenia on abdominal imaging are essential for detecting ICA even in the absence of any family history. After the diagnosis of ICA, patient and parent education, vaccinations, antibiotic prophylaxis, and prompt empiric treatment of febrile episode should be provided.

## Background

The spleen is critical in the predominantly innate immune response to infections from encapsulated bacterial pathogens such as *Streptococcus pneumoniae* [1]. Congenital asplenia often occurs as part of a recognized malformation syndrome, with anomalies of the heart, great vessels, and viscera [2]. The best known among these syndromes is the congenital asplenia/polysplenia syndrome associated with visceroarterial heterotaxy; its incidence is estimated at approximately 1/10,000 to 1/40,000 live births [3]. In contrast, isolated congenital asplenia (ICA) occurs less frequently. A recent French nationwide study indicated that the prevalence of ICA is 0.51 per million births [3]. Patients with congenital asplenia have increased susceptibility to invasive infections along with a high mortality rate despite aggressive therapy. Although the initial symptoms may be mild and nonspecific, it can progress rapidly to Waterhouse-Friderichsen syndrome with full-blown septic shock and disseminated intravascular coagulation (DIC). Compared to congenital asplenic syndrome, the diagnosis of ICA is more difficult due to the lack of other anomalies. In particular, in sporadic ICA, the condition may remain undetected until the postmortem autopsy.

Here, we present the case of a girl with sporadic ICA who developed overwhelming meningitis due to *S. pneumoniae* and compared the clinical manifestations between sporadic and familial ICA cases by reviewing the literature.

## Case presentation

A previously healthy 11-month-old female infant (the first child of non-consanguineous Japanese parents) was referred to our hospital with clinical signs of septic shock and purpura, which were preceded by fever for 2 days and signs of upper respiratory tract infection (URI) for 1 week. She had previously received oral antibiotic treatment for the URI. The patient had not received pneumococcal or *Haemophilus influenzae* type b (Hib) vaccination because these vaccines were not included in the routine immunization program at that time in Japan. On admission, she had a body temperature of 39.4 °C, heart rate of 180 bpm, blood pressure of 83/46 mmHg, and cold extremities with a capillary refill of >5 s. She was irritable without any focal neurological deficits. She was found to have purpura fulminans with ecchymoses of the face and trunk and purpura of her limbs and ears. Her neck was stiff. Laboratory examination indicated a white blood cell count of 35.1 × 10^9^/L, with 67% neutrophils and 25% lymphocytes; platelet count of 8.1 × 10^9^/L; hemoglobin level of 10.9 g/dL; and C-reactive protein level of 18.5 mg/dL. Prolonged prothrombin time and activated partial thromboplastin time and elevated levels of plasma fibrinogen degradation products (235 μg/mL: normal <5 μg/mL) indicated the presence of DIC. A lumbar puncture indicated the presence of pleocytosis in the cerebrospinal fluid (CSF), including 11,527 cells/μL (95% polymorphonuclear cells), 11 mg/dL glucose (blood glucose level; 87 mg/dL), and 425 mg/dL protein. The gram stains of CSF revealed the presence of gram-positive diplococci. We diagnosed the patient with bacterial meningitis followed by DIC and treated her with intravenous antibiotics (ampicillin and cefotaxime) empirically and addressed the DIC using anticoagulants. On the day after admission, blood and CSF cultures indicated positive results for *S. pneumoniae* (serotype not available). The minimum inhibitory concentration for penicillin was <0.06 μg/mL. The patient received antibiotic therapy for 14 days with a good clinical response and was discharged without any sequelae.

**Fig. 1:**
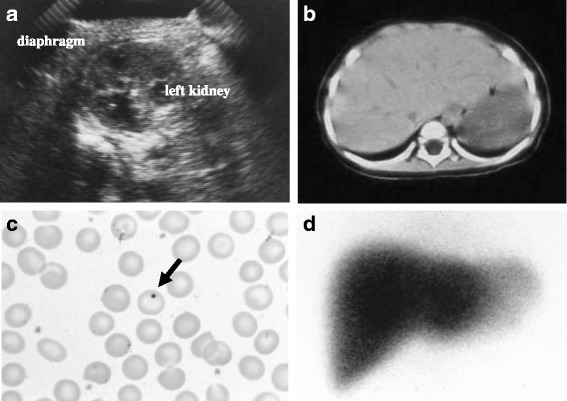
**a** Ultrasonography of the abdomen indicating the absence of a spleen; the upper pole of the left kidney is located beneath the diaphragm. **b** Nonenhanced computed tomography of the abdomen indicating the lack of a spleen on the left. **c** Peripheral blood smear showing the presence of Howell-Jolly body (arrow). **d** Scintigraphy with Tc-99 m phytate colloid showing no functional spleen tissue

Over the clinical course, abdominal ultrasonography (US) was performed to detect adrenal hemorrhage as we suspected Waterhouse-Friderichsen syndrome; however, US did not indicate the presence of a spleen (Fig. 1a). In addition, no spleen was visible on abdominal computed tomography (Fig. 1b). Careful blood smear assessment indicated the presence of Howell-Jolly bodies (HJB) (Fig. 1c). Based on the suspicion of asplenia, scintigraphy with Tc-99 m phytate colloid was performed and no functioning splenic tissue was detected (Fig. 1d). The patient had no history of splenectomy. No other malformations such as heart defects, anomalous systemic or pulmonary venous connections, and abdominal heterotaxia were detected, leading to the diagnosis of ICA. Moreover, peripheral blood smears and spleen US examinations of the parents indicated normal results. After that, antibiotic prophylaxis with amoxicillin was initiated as well as vaccinations using 7-valent pneumococcal conjugate vaccine (PCV7) and Hib vaccine, followed by 23-valent pneumococcal polysaccharide vaccine (PPSV23) when she was 2 years old. The parents were counseled about the need for prompt medical attention after the onset of fever. Subsequently, the patient has not experienced any invasive infections for >5 years.

## Discussion

The congenital absence of the spleen has been recognized as a condition for more than 270 years. The first two cases, reported in 1740, were observed independently by Pohl and Jauch; in each of these young adult male cases, absence of the spleen was noted on autopsy [4]. Putschar and Manion described 11 cases (including the two mentioned above) of spleen agenesis without any other malformations in 1956 [4]; to our knowledge, that is the first series on ICA and 70 such patients have been reported to date [5].

In congenital asplenic syndrome, different modes of inheritance have been reported; mostly autosomal recessive, exceptionally autosomal dominant or X-linked. Two human genes are reportedly involved in this syndrome, connexin 43 and *ZIC3*, which are involved in an X-linked form [6]. On the other hand, most of the reported ICA cases are familial and the mode of inheritance is usually autosomal dominant, although recessive inheritance patterns have also been observed [3, 6]. Given the isolated abnormality of spleen development, mutations in the *Hox 11* have been reported in mice with ICA [7]. Moreover, a recent study in humans with autosomal dominant ICA demonstrated that germline mutations in *RPSA* lead to a spleen-specific developmental defect [8]. However, the genetic etiology of ICA remains unknown. With respect to sporadic ICA cases, Gilbert et al. described 13 of 31 cases of ICA between 1956 and 2000 [6], and Mahlaoui et al. documented 29 of 65 cases between 1956 and 2009 [3]; these researchers speculated that sporadic cases might be related to either the low penetrance of ICA or de novo mutations. In the present report, we reviewed 73 cases with ICA in the literature between 1956 and 2016 using the Medline database and identified 32 sporadic cases [3, 5, 9–27]. Their clinical details are summarized in Table 1. The first sign of ICA was severe invasive bacterial infection in 25 of 32 cases (78%), and 12 patients died. Of the 25 patients, only four had multiple episodes of invasive infections, including two episodes in three cases (two deaths) and three episodes in one case (no death). In a total of 29 episodes of overwhelming sepsis, the causative pathogen was *S. pneumoniae* in 18 episodes (62%), *H. influenzae* in six episodes (21%), and *Escherichia coli* in two episodes (7%). Of the 12 patients who died, at least nine (75%) died within the first 48 h of hospital admission and seven (58%) died within the first 24 h. The mortality rate for overwhelming postsplenectomy infection (OPSI) has been estimated as approximately 50–70% despite aggressive therapy [28], and >50% died within the first 48 h of hospital admission [29]. Similar to OPSI, overwhelming infection in ICA is a virulent entity that leads to death within 48 years in most cases. Hence, the diagnostic workup should never delay the initiation of empiric antibiotic therapy [30]. A delay of only a few hours may lead to death. Moreover, overwhelming infection in asplenia may also occur at any time after birth and should also be considered in adults, as demonstrated in our case series. As individuals who have undergone splenectomy can be identified as having infection by themselves or by clinicians, some OPSI cases may still be preventable. When asplenia develops as part of heterotaxy syndrome, it is often identified due to the presence of other congenital anomalies, and appropriate management may be instituted to prevent severe infection. However, in the case of ICA, there are no other physical clues for diagnosis and overwhelming infection may be the first sign of the disease [9–11]. In the 73 cases available for review, we compared the clinical characteristics between sporadic and familial ICA using the Mann-Whitney U-test (the Statistical Package for Social Sciences version 23, Tokyo, Japan) (Table 2). The analysis showed that event-free cases were significantly more common in familial ICA (*p* = 0.001). In a familial case, the identification of other family members with the condition may provide an important clue for the diagnosis. However, in sporadic cases, the condition may remain undetected even at the presentation with pneumococcal disease. In the present case, the diagnosis of asplenia in the context of sepsis was confirmed through US of the abdomen and identification of HJB on blood smears. In the case series, HJBs were identified in at least 18 patients (60%). Abdominal US and HJB assessment are important tests to perform in the first-degree relatives of affected individuals for the diagnosis of asplenia.

**Table 1:** Reported cases of sporadic congenital isolated asplenia

Case no.	Age at diagnosis	Sex	Clinical events	Pathogens	Howell-Jolly bodies	Outcome	Reference
1	2 months	Female	Meningitis	*H. influenzae*	Yes	Survived	[13]
2	4 months	Unknown	Sepsis	*E. coli*	NA	Died	[11]
3	6 months	Unknown	Sepsis	*S. Pneumoniae*	NA	Survived	[11]
4	6 months	Female	Sepsis, purpura fulminans	Unknown	Yes	Survived	[3]
5	7 months	Male	Sepsis, bilateral adrenal hemorrhage	*H. influenzae*	NA	Died in 12 h	[15]
6	7 months	Female	Asymptomatic	No infection	Yes	Alive	[3]
7	10 months	Unknown	Sepsis	*H. influenzae*	NA	Survived	[11]
8	10 months	Male	Sepsis, purpura fulminans	*S. pneumoniae*	Yes	Died in 20 h	[3]
9	11 months	Female	Meningitis, purpura fulminans	*S. pneumoniae*	Yes	Survived	Present case
10	12 months	Female	Sepsis, purpura fulminans	*S. pneumoniae*	Yes	Survived	[3]
11	12 months	Male	Meningitis (twice), osteomyelitis	*S. pneumoniae*	Yes	Survived	[14]
12	20 months	Female	Sepsis	Unknown	Yes	Survived	[3]
13	21 months	Unknown	Sepsis	*S. pneumoniae*	NA	Survived	[11]
15	1 years	Unknown	Sepsis	*S. pneumoniae*	NA	Survived	[27]
16	2 years	Male	Sepsis, bilateral adrenal hemorrhage	*H. influenzae*	NA	Died in 32 h	[15]
17	2 years	Female	Meningitis (3 times)	*E. coli, S. pneumoniae*	Yes	Survived	[5]
				*H. influenzae*			
18	3 years	Unknown	Sepsis	*H. influenzae*	NA	Died in 24 h	[11]
19	4 years	Female	Sepsis, bilateral adrenal hemorrhage	*S. pneumoniae*	NA	Died in 12 h	[18]
20	5 years	Male	Sepsis, meningitis	*S. pneumoniae*	NA	Died	[23]
21	10 years	Female	Sepsis, arthritis (twice)	*S. pneumoniae*	NA	Died in 24 h	[21]
22	15 years	Male	Meningitis (twice), bilateral adrenal hemorrhage	*S. pneumoniae*	Yes	Died in 3 h	[12]
23	28 years	Male	Sepsis	Group B *streptococcus*	Yes	Survived	[24]
24	20 years	Male	Tonsillitis	Unknown	Yes	Alive	[22]
25	36 years	Male	Meningitis, bilateral adrenal hemorrhage	*S. pneumoniae*	NA	Died in 29 h	[10]
26	37 years	Female	Thrombocytosis	No infection	Yes	Alive	[16]
27	44 years	Female	Thrombocytosis	No infection	Yes	Alive	[25]
28	56 years	Female	Thrombocytosis	No infection	Yes	Alive	[20]
29	56 years	Male	Thrombocytosis	No infection	Yes	Alive	[20]
30	60 years	Female	Meningitis	*S. pneumoniae*	Yes	Survived	[19]
31	67 years	Female	Sepsis	*S. pneumoniae*	NA	Died in 24 h	[26]
32	72 years	Male	Thrombocytosis	No infection	Yes	Alive	[28]

*NA* Not available

**Table 2:** Comparison of clinical characteristics between sporadic and familial cases of isolated congenital asplenia

	Sporadic *n* = 32	Familial *n* = 41	*P* value
Age at diagnosis, years	2.1 (0.8–34.2)	1.0 (0.6–3.0)	0.04
Male sex	11^a^ (44)	25(61)	0.18
Clinical events			
Sepsis and/ or meningitis	25(78)	24 (58)	0.08
Others	6 (19)	2 (5)	0.06
Asymptomatic	1 (3)	15 (37)	0.001
Pathogens			
S. pneumoniae	16^b^ (62)	14^c^ (56)	0.69
H. influenzae	5^b^ (19)	3^c^ (12)	0.48
Others	3^b^ (12)	0^c^ (0)	0.08
Howell- Jolly bodies	18 (56)	17 (41)	0.21
Outcome			
Death	12 (38)	16 (39)	0.68

Values are presented as median (interquartile range) or number (percentage). *P* < 0.05 is considered to be significant. ^a^data from 25 patients were available. ^b^data from 26 patients were available. ^c^data from 25 patients were available

As for post-diagnosis management, immunization against encapsulated bacteria is an indispensable strategy to ensure the survival of asplenic patients. Moreover, PCVs enhance immunity in asplenic patients because PCVs can induce antibody responses in the absence of a spleen [31, 32]. Therefore, in ICA cases, the use of active immunizations including pneumococcal (not only PPSV but also PCV), Hib, and meningococcal vaccines could prevent overwhelming sepsis and save lives [33, 34]. In Japan, Hib conjugate vaccine has been available on a voluntary basis since 2008, and PCV has been available since 2010; however, the vaccination rate was estimated to be <10% in those years [35]. Subsequently, these two vaccines have been included in the routine immunization program since 2013. In fact, the present patient did not receive the PCV or Hib vaccine before the septicemic onset because these vaccinations were voluntary at the time. Moreover, meningococcal vaccine has not been approved yet in Japan. In contrast, in Western countries, greater efforts, including the publication of national guidelines, have been made to improve the management of asplenic patients [33, 34]. As for the latest recommendations, asplenic patients should receive vaccines including 13-valent PCV (PCV13) and Hib vaccine for children aged <2 years as recommended routinely for immunocompetent persons based on the Centers for Disease Control and Prevention annual schedule, followed by PPSV23 at 2 years of age or older, with a recommendation for a second dose of PPSV23 5 years later. Moreover, some European public health agencies recommend PPSV23 reimmunization of asplenic individuals every 5 years [36]. Meningococcal vaccine should also be administered to asplenic patients aged ≥2 months. In addition, yearly influenza vaccination is recommended for all asplenic patients aged >6 months because the influenza virus is an important risk factor for secondary pneumococcal and *H. influenzae* infections [34]. However, vaccination does not fully eliminate the risk of invasive infection [32]. The second major strategy is antibiotic prophylaxis and treatment. Daily antibiotic prophylaxis against pneumococcal infections is recommended due to evidence of good efficacy in patients with sickle cell anemia (functional asplenia) [37, 38]. However, the appropriate duration of prophylaxis in asplenic patients is unknown. The use of life-long penicillin prophylaxis has potential disadvantages as it can be associated with the development of bacterial resistance, may have side effects including allergy, and may be associated with poor adherence [39]. The American Academy of Pediatrics recommends prophylaxis until the child is 5 years of age [38]. In contrast, British guidelines recommend life-long penicillin prophylaxis in high-risk patients defined as those <16 or >50 years of age, those with an inadequate serological response to pneumococcal vaccination, and those who have had a previous episode of invasive pneumococcal disease [34]. Education for asplenic patients and their parents is also important to enable them to understand that all febrile illnesses are potentially serious in asplenic patients and that patients developing such signs must be given systemic antibiotics and admitted to hospital. If an asplenic patient travels to or resides in an area where medical care is not accessible, an appropriate antibiotic agent should be readily available and the child’s caregiver should be instructed in its appropriate use [38]. Patients and parents should also be advised about animal handling as overwhelming sepsis (due to *Capnocytophaga canimorsus*) may result from dog, cat, or other animal bites [40]. Furthermore, these patients require specific advice regarding travel as they are at increased risk of severe malaria (mosquito bites) and babesiosis (tick bites) in endemic areas [34, 38].

## Conclusions

In the case of severe invasive pneumococcal disease, a systematic search for HJB on blood smears and imaging examination of the abdomen for the presence of asplenia are vital for detecting ICA, even in the absence of a family history. If asplenia is detected, life-saving vaccination with a series of PCV13 and Hib vaccines, followed by PPSV23, in addition to penicillin prophylaxis, preemptive investigation, and empiric treatment early in the course of illnesses should be provided.

Considering the limitations of the literature review, this article may have incomplete data about the full syndrome of asplenia. The inclusion of unwritten, unpublished, or repetitive cases, or cases published in grey literature might have influenced the findings.

## Abbreviations

CSF: Cerebrospinal fluid
DIC: Disseminated intravascular coagulation
Hib: *Haemophilus influenzae* type b
HJB: Howell-Jolly bodies
ICA: Isolated congenital asplenia
OPSI: Overwhelming postsplenectomy infection
PCV7: 7-valent pneumococcal conjugate vaccine
PPSV23: 23-valent pneumococcal polysaccharide vaccine
URI: Upper respiratory tract infection
US: Ultrasonography
